# Digital Image Sensor-Based Assessment of the Status of Oat (*Avena sativa* L.*)* Crops after Frost Damage

**DOI:** 10.3390/s110606015

**Published:** 2011-06-03

**Authors:** Antonia Macedo-Cruz, Gonzalo Pajares, Matilde Santos, Isidro Villegas-Romero

**Affiliations:** 1 Colegio de Postgraduados, Campus Montecillo, km. 36.5 carretera México-Texcoco, cp 56230, Montecillo, Texcoco, Estado de México, C.P. 56230, México; 2 Facultad de Informática, Universidad Complutense de Madrid, 28040-Madrid, Spain; E-Mails: pajares@fdi.ucm.es (G.P.); msantos@dacya.ucm.es (M.S.); 3 Universidad Autónoma Chapingo, km 38.5 carretera México-Texcoco, cp 56230, Chapingo, Texcoco, Estado de México, C.P. 56230, México; E-Mail: isidrovr@colpos.mx (I.V.-R.)

**Keywords:** digital image sensor, agricultural images, unsupervised classification, automatic thresholding, CIELab colour space, fuzzy error matrix, oat frost damage

## Abstract

The aim of this paper is to classify the land covered with oat crops, and the quantification of frost damage on oats, while plants are still in the flowering stage. The images are taken by a digital colour camera CCD-based sensor. Unsupervised classification methods are applied because the plants present different spectral signatures, depending on two main factors: illumination and the affected state. The colour space used in this application is CIELab, based on the decomposition of the colour in three channels, because it is the closest to human colour perception. The histogram of each channel is successively split into regions by thresholding. The best threshold to be applied is automatically obtained as a combination of three thresholding strategies: (a) Otsu’s method, (b) Isodata algorithm, and (c) Fuzzy thresholding. The fusion of these automatic thresholding techniques and the design of the classification strategy are some of the main findings of the paper, which allows an estimation of the damages and a prediction of the oat production.

## 1. Introduction

Oat *(Avena sativa* L.*)* is one of the most cropped cereals in the World [1], with an annual production of 26 million tons of grain [2]. In Mexico, oats are cultivated on 810,412 ha. During the growing season 2005 and 2009 frost damage affected an area of 76,166 ha [3,4] causing substantial losses.

The degree of tolerance shown by a plant to freezing depends largely on the stage of development at which the stress occurs. Before the initiation of flowering, usually 8–10 weeks after germination, cereal plants are capable of withstanding extreme cold. But the most susceptible developmental stage to frost damage is the period from pre-heading to flowering, after ear emergence. The reaction of the oat plant to frost then changes markedly. To sum up the effects of frost damage on cereals: during and after ear emergence plants become very susceptible to frost injury. Frost damage after head-emergence often causes severe stem and head damage. Also, damaged tissues develop a water-soaked dark green colour (a bit like frozen lettuce) and later dry out and bleach. Therefore, the connection between the head and the rest of the plant is affected, and the head dies.

In Mexico, frost between October and December (10 to 60 days), with minimum temperatures as low as −13 °C, can damage substantially the oat crops. Freezing injury of leaves occurred over the range of −2 °C to −4 °C in both the winter and spring cultivars of oat [5].

The estimation of the damage is of great interest because it would allow a more accurate forecast of the crop production. This paper is focused on the estimation of oat crop losses to frost, *i.e.*, produced by freezing temperatures. The proposal can also be applied to damages caused by other factors (including plant disease, hail, drought ...), and it can be useful in future to find specific treatments in order to control pests, diseases and other harmful agents.

Spatial analysis tools are currently used throughout agriculture, livestock and forestry with different specific objectives [6,7]. In this sense, the images acquired by remote sensors provide the necessary spatial resolution to obtain information about objects, areas, or phenomena on the earth’s surface, at different scales. These sensors measure the intensity of the energy emitted or reflected by objects using the electromagnetic spectrum [8]. One of the most important medium and large term applications is the generation of thematic maps, where each pixel of a given image is labelled by a classification rule, which specifies the type of object that exists in the reference zone [9]. Nevertheless, remote sensors are expensive and images are not always easy to obtain. The use of conventional digital camera in spite of other sensors has the advantages of providing the necessary spatial information for the analysis while digital images are easy to be obtained and more accessible. They have been proved successful for classification purposes in the agriculture field, such as for the identification of weeds [10,11], and to detect obstacles in the operation of automated farm machinery [12]. There are many approaches to the identification of textures in agricultural images. Most of them can be grouped as follows.

1. Visible spectral indices for identifying green plants, including crops and weeds [13–16]. These methods, some of them automatic, are based on greenness (plants and weeds) and redness (soil, stones, debris, *etc*.) identification.
2. Specific histogram threshold-based approaches, including dynamic thresholding. A global thresholding technique is one that thresholds the entire image with a single threshold value; a local thresholding technique is one that first partitions a given image into subimages and then determines a threshold value for each of these subimages, whereas a dynamic thresholding technique assigns a possibly different threshold value to each pixel in the image [17]. In [18] a decision function is estimated under the assumption that the two classes follow Gaussian distributions. Otsu’s method [19] has been applied to gray images considering a bi-class problem [20,21]. In [22], a thresholding approach is applied to images previously transformed from RGB to gray scale. This method was later improved using local homogeneity and morphological operations in [23]. In [24], the authors apply a combination of greenness and intensity derived from the red and green spectral bands; they determine an automatic threshold for a bi-class problem assuming two Gaussian probability density functions associated to soil and vegetation, respectively. In [16], the automatic Otsu histogram thresholding method is applied for binarizing the image once the greenness is extracted, and then the normalized difference index (NDI) is obtained. After different experiments, the authors conclude that a threshold of zero is enough for the proposed application, and therefore in the end Otsu’s method was not applied in that work.
3. Learning-based methods. In [25], fuzzy clustering partitions images into regions of interest based on the greenness and redness. The environmentally adaptive segmentation algorithm (EASA) proposed in [26] is based on its adaptability for detecting green plants through a supervised learning process. This method was tested in [27], using the HSI (hue-saturation-intensity) colour space to deal with the illumination variability. The mean shift algorithm was applied in [28], on the assumption that the segmentation of vegetation and background can be considered as a bi-class problem; the separation of classes was validated using neural networks and the Fisher linear discriminant; the colour spaces used were RGB, LUV and HIS. In remote sensing, unsupervised approaches have been designed for hyper-spectral images [29,30], where each pixel is supposed to be a linear combination of spectral signatures of the hyper-spectral space. In [31], an automatic strategy is designed for remote sensing images classification in natural images based on Otsu’s histogram thresholding method.

Based on these considerations and to address the classification problem presented in this paper, a new automatic strategy has been designed according to the following guidelines:

- Oat crop images can present very different spectral signatures due to several factors that can cause damage. On the other hand, supervised approaches cannot be appropriately trained as texture patterns are not known in advance. Therefore, an unsupervised automatic classification has to be designed.
- Coverage becomes irrelevant when dealing with oat crops affected by frost, since the ground surface is usually completely covered by plants. Hence, methods based on computation of vegetation indices such as some of the mentioned are infeasible or not suitable. Besides, those approaches require setting a threshold for final segmentation, in contrast to the automatic procedure proposed in this paper where thresholds are automatically found.
- Automatic histogram thresholding-based approaches appear as promising techniques in bi-class classification problems. Their extension for solving unsupervised multi-classification tasks with acceptable results, as in [10], encourages us to apply it.
- The CCD sensor of the digital camera captures images of the crop fields in outdoor environments, *i.e*., with high illumination variability. The sensor response is proportional to the light energy projected onto its surface. This energy depends on the visible wavelengths reflected by the objects (plants and soil). Each wavelength produces a different response which is assigned into a standard colour.

The CIELab colour model [32] is less illumination-dependent. CIELab defines colors more closely to the human color perception [33], and according to [34], this colour model is considered approximately uniform, *i.e.*, the distance between two colours in a linear colour space corresponds to the differences perceived between them. The CIELab color space is based on the concept that colors can be considered as combinations of red and yellow, red and blue, green and yellow, and green and blue. To determine the exact combination of colors of a product, coordinates of a three dimensional color space are assigned (L*, a*, b*). The three color coordinates are the lightness, the red/green and the yellow/blue coordinate respectively. More details will be given in Section 2.3.

Different histogram thresholding approaches have been presented in the literature [17]. In [35], an evaluation of seven automatic thresholding algorithms has been carried out on images with high variability as in this classification problem. The analysed methods are: (1) Isodata algorithm [36]; (2) Otsu’s algorithm [19]; (3) Minimum error thresholding [37]; (4) K-means clustering algorithm [38]; (5) Entropic of the histogram [39]; (6) Moment preserving method [40]; and (7) Fuzzy thresholding [41]. According to [35], the best performances are obtained by Isodata, Otsu, Fuzzy thresholding and Moment preserving, being Isodata and Moment preserving quite similar. Based on this study, the three first ones have been selected to be conveniently combined in the proposed method. The synergy of those strategies is one of the main contributions of this paper as it improves the segmentation of agricultural images.

Automatic successive thresholding is applied to each one of the three spectral bands of the CIELab colour space, L*, a* and b*, allowing the partition of these histograms into several regions. The combination of these regions determines different classes where each pixel can be classified into. Depending on the nature of the problem the number of regions varies, but they must be enough to cope with the classification problem. Thanks to this flexibility, the proposed strategy becomes unsupervised with a variable number of clusters.

The rest of the paper is organized as follows. In Section 2, materials and methods are described. It includes digital image processing, sampling method, colour space transformation, the new combined thresholding approach, and the design of the unsupervised classification strategy. In Section 3, classification results are presented and discussed. Conclusions end the paper.

## 2. Materials and Methods

### 2.1. Digital Images

The material used to test the proposal is the following. Digital photographs of oats in the flowering stage were taken 15 days after the last frost (November 2010, Mexico). The image sensor type was CCD 1/2.3″; focal length: 35 mm, Canon digital IXUS 85 IS, resolution 3,648 × 2,736 pixels. The digital image sensor was previously calibrated in order to estimate the intrinsic (focal length and radial distortion) and extrinsic parameters (translation displacements and rotation angles) [42]. This is required for the determination of the surface that is imaged.

### 2.2. Sampling Method

The whole oat crop population is split into two regions, according to the affectation degree: a highly affected by frost region (nearly dried) and a lower affected one. For each of these two regions, a random sampling is applied. It consists of taking 2,000 samples of a total surface of 20 ha, *i.e*., 100 samples/ha (50 images of the most affected area and 50 of the healthy area per hectare). Global Positioning System (GPS) measurements with the Universal Transverse Mercator (UTM) coordinate system were applied to ensure that samples do not overlap, and that they are significant enough to assure a high coverage of the crop surface. Four different GPS measurements o the distance to the centre of the sample were averaged. This value is assigned to the central pixel of the image. Accurate GPS measurements are not critical in this application.

**Figure 1.**
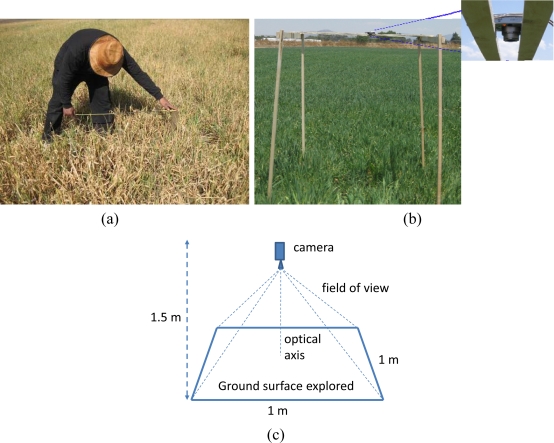
**(a)** and **(b)** Selection and sample delimitation of the oat crop to be photog aphed; **(c)** system geometry with the optical axis perpendicular to the ground.

Each sample was conveniently measured and posted. Figure 1(a) shows this process in a highly damaged field. Once the sample is selected, the camera is placed with its optical axis perpendicular to the ground and at a height of 1.5 m. Figure 1(b) displays the setup in a healthy oat area. Figure 1(c) shows the ground surface explored by the projection of the camera view. The posting process is required for the verification process (see Section 3.2) and also for future analysis of the development of the affected crops.

### 2.3. Colour Image Pre-Processing

The images are originally captured in RGB colour model. Nevertheless, as it was explained in the introduction, the CIELab colour model (CIE L*a*b*) [32] was selected as the most appropriate to deal with high illumination variability. CIE is the French abbreviation of the International Commission of Light (Commission Internationale d'Eclairage). The L* component represents the perceived lightness, and the other two, a* and b*, are the Chroma components (colour-opponent dimensions), a set of contrasting colour axes (red-green, and yellow-blue). The images are therefore transformed from RGB to CIE L*a*b* colour model by the mathematical transformation given by Equation (1):

(1) $$\left[\begin{array}{c} X\\ Y\\ Z \end{array}\right]=\left[\begin{array}{ccc} 0.490 & 0.310 & 0.200\\ 0.177 & 0.813 & 0.011\\ 0.000 & 0.010 & 0.990 \end{array}\right]\left[\begin{array}{c} R\\ G\\ B \end{array}\right]$$

In the new space X, Y, Z, the lightness is calculated by Equation (2):

(2) $$\begin{array}{l} L^{*}=\{\begin{array}{cc} 116{\left(\frac{Y}{Y_{n}}\right)}^{1/3}-16 & \text{if}\;\frac{Y}{Y_{n}}>0.008856\\ 903.3{\left(\frac{Y}{Y_{n}}\right)}^{1/3} & \text{otherwise} \end{array} \end{array}$$

The a* and b* components are obtained by:

(3) $$\begin{array}{l} a^{*}=500\left[f\left(\frac{X}{X_{n}}\right)-f\left(\frac{Y}{Y_{n}}\right)\right]\\ b^{*}=200\left[f\left(\frac{Y}{Y_{n}}\right)-f\left(\frac{Z}{Z_{n}}\right)\right] \end{array}$$

where *f*(*t*) = *t^1/3^* for *t >* 0.008856, and *f*(*t*) = 7.787 *t +* 16/116 otherwise. Normalized *X_n_*, *Y_n_*, *Z_n_*_,_ are the CIE XYZ tristimulus values of the reference white point (amount of three primary colours in a three-component additive colour model). The division of the *f*(*t*) function into two domains was done to prevent an infinite slope at *t* = 0; *f*(*t*) was assumed to be linear below some *t = t_0_*, and it was also assumed to match the *t^1/3^* part of the function at *t_0_* in both, value $t_{0}^{1/3}=at_{0}+b$, and slope $1/(3{\text{t}}_{0}^{2/3})=a$. The value of b was chosen to be 16/116 as usual.

### 2.4. The Proposed Combined Thresholding Approach

Once the image has been transformed into the CIELab colour space, three components are now available: L*, a* and b*. For each component, a threshold is automatically computed based on the combination of the three following thresholding methods: Isodata, Otsu and Fuzzy. Some details about each method are briefly presented.

#### 2.4.1. Isodata Thresholding

The Iterative Self-Organizing Data Analysis Technique (Isodata) is a simple iterative technique for choosing a threshold developed in 1978 [36]. The objective of the Isodata algorithm is to split non-homogeneous regions into two sub-regions (objects and background). According to [35], initially a guess is made at a possible value of a threshold. Then, the mean values of the two categories (objects and background) produced with this threshold are estimated. The threshold is moved to the middle of the distance between the two mean values. The procedure is repeated again and a new threshold is obtained. The process continues until the threshold stops changing its value.

Let the histogram of pixel values be denoted by *h*(0), *h*(1),…, *h*(*L* − 1), where *h*(*i*) specifies the number of pixels of an image whose greyscale value is *i*, and *L* − 1 is the maximum pixel greyscale value of the image. The initial guess at *t_i_* is set to the mean value. Then, for smaller or equal values than it, *t* ≤ *t_i_*, the average, *μ*_1_(*t*), is computed; otherwise, *μ*_2_(*t*) is calculated Equation (4):

(4) $$\mu_{1}(t)=\sum_{i=0}^{t}i\cdot h(i)/\sum_{i=0}^{t}h(i)\;\;\;\;\;\;\;\;\;\;\mu_{2}(t)=\sum_{i=t+1}^{L-1}i\cdot h(i)/\sum_{i=t+1}^{L-1}h(i)$$

The *t_i_* value is re-estimated as the integer part of the mean value of *μ*_1_ and *μ*_2_, until *t_i_* stops changing. Then the last threshold value is renamed as *t*_I_.

#### 2.4.2. Otsu’s Method

Otsu’s method is one of the most popular techniques of optimal thresholding, based on discriminant analysis [19]. It maximizes the between-class variance of the histogram, $\sigma_{B}^{2}(t)$, and gives the best separation of classes in an image. Let the pixels of a given image be represented in *L* grey levels, [0, 1, 2, …, *L* − 1]. The number of pixels at level *i* is denoted by *h*(*i*), and the total number of pixels by *N*. The grey level histogram is normalized and considered as a probability distribution. The probability of occurrence of each grey level *p*(*i*) is then Equation (5):

(5) $$p(i)=\frac{h(i)}{N},\;\;\;p(i)\geq 0,\;\;\;\;\sum_{i=0}^{L-1}p(i)=1$$

The zero-th *w*(*t*) (accumulated probability), the first-order cumulative moments of the histogram up to the *t-*th level, *μ*(*t*), and the total mean level of the image, *μ_T_*, are obtained by Equation (6):

(6) $$w(t)=\sum_{i=0}^{t}p(i);\;\;\;\;\;\;\;\;\;\mu (t)=\sum_{i=0}^{t}i\cdot p(i);\;\;\;\;\;\;\;\;\;\;\;\;\mu_{T}=\sum_{i=0}^{L-1}i\cdot p(i)$$

The optimal threshold *t_o_* is then the value that maximizes Equation (7):

(7) $$\sigma_{B}^{2}(t_{O})=\text{max}\;\sigma_{B}^{2}(t)\;\;\;\;\text{where}\;\;\;\;\sigma_{B}^{2}(t)=\frac{{\left[\mu_{T}w(t)-\mu (t)\right]}^{2}}{w(t)[1-w(t)]}$$

#### 2.4.3. Fuzzy Thresholding

This algorithm is based on the fuzzy set theory and it was proposed in [41]. It makes a partition of the image space by minimizing the measure of its fuzziness. This measurement can be expressed by different functions, one of them the entropy. The membership function, *μ_F_*(*I*(*x*, *y*)), can be viewed as a characteristic function that represents the fuzziness of a given pixel (*x*,*y*) of the *M* × *N* image *I*:

(8) $$\mu_{F}(I(x,\;y))=\{\begin{array}{cc} \frac{1}{1+|I(x,\;y)-\mu_{1}(t)|} & \text{if}\;\;I(x,\;y)\leq t\\ \frac{1}{1+|I(x,\;y)-\mu_{2}(t)|} & \text{if}\;\;I(x,\;y)>t \end{array}$$

where *μ*_1_(*t*) and *μ*_2_(*t*) have been already defined in Equation (4). According to [35], the entropy of an image *I*, named *E*(*I*), calculated by Equation (9), has been chosen as the measure of fuzziness by means of the Shanon’s function (10):

(9) $$E(I)=\frac{1}{MN\;\text{ln}2}\sum_{i=0}^{L-1}S(\mu_{F}(i))h(i)$$

(10) $$S(\mu_{F}(i))=-\mu_{F}(i)\;\text{ln}\;[\mu_{F}(i)]-[1-\mu_{F}(i)]\text{ln}[1-\mu_{F}(i)]$$

The optimal threshold, *t_F_*, can be estimated by minimizing the measure of fuzziness *E*(*I*) as follows Equation (11):

(11) $$t_{F}=\text{arg}\;\text{min}\;E(I)$$

#### 2.4.4. Combination of Thresholds

For each of the three spectral component in the CIE colour space, three different thresholds, *t_I_*, *t_o_* and *t_F_*, are obtained as the result of applying the three thresholding algorithms. Several combinations derived from the classification theory can be applied [43] to find a unique threshold value in order to improve the results of the classification. The average, *i.e.*, *t* = (*t_I_* + *t_O_* + *t_F_*)/3, is the simplest function. One advantage of the average is that the highest and lowest values of the threshold are smoothed. The combined threshold is denoted *t_L_*, *t_a_*, and *t_b_*, for each spectral component, L*, a* and b*, respectively. Then, each spectral channel is partitioned into two regions by its corresponding threshold.

### 2.5. Unsupervised Classification Strategy

There are three steps in the proposed classification strategy. First, the assignment process, that consists in assigning one of the possible classes to each pixel. Second, the codification of each cluster, which is identified by a label. Finally, a regrouping process so that very similar classes are merged into one.

#### 2.5.1. Assignment Process

Given a pixel *i* located at (*x*, *y*) in the original RGB image, it is transformed to the CIE L*a*b* colour space. Its three spectral components in this space are obtained, namely L*(*x*, *y*) = *i_L_*, a*(*x*, *y*) = *i_a_* and b*(*x*, *y*) = *i_b_*.

As already mentioned, the thresholding methods split the histogram into two regions. As there are three spectral components, six sub-regions are obtained. If necessary, successive thresholding can be applied to each spectral channel. The second thresholding produces three partitions per channel. If a third thresholding is applied, four regions per component are obtained and so on. Therefore, assuming that eventually the number of thresholds per channel is *M*, there will be *t_L_*_1_, *t_L_*_2,_ … *t_LM_*, thresholds for channel L*, and in the same way, *t_a_*_1_, *t_a_*_2_, …, *t_aM_* for component a*, and *t_b_*_1_, *t_b_*_2_, …, *t_bM_*, for component b*. Based on this, each pixel *i* can be coded as $\tilde{\iota_{s}}$ according to its spectral components by Equation (12):

(12) $${\tilde{i}}_{s}\{\begin{array}{lll} 0 & \text{if} & i_{s}\leq t_{s1}\\ 1 & \text{if} & t_{s1}<i_{s}\leq t_{s2}\\ 2 & \text{if} & t_{s2}<i_{s}\leq t_{s3}\\ & & \;\;\;\;\;\;\vdots \\ M & \text{if} & i_{s}>t_{sM} \end{array}$$

where *s* denotes the spectral component, *i.e.*, *s* = L, a or b, and *t_si_* are the consecutive thresholds.

For example, it is known that in the CIE L*a*b* colour space values for L* are in the range [0, 100] while a* and b* are in the interval [ 110, 110]. So, considering the spectral component a* with two thresholds, *t_a_*_1_ = 20 and *t_a_*_2_ = 60, a pixel will be coded as 0, 1, or 2, if its spectral value a* is smaller than 20, between 20 and 60, or greater than 60, respectively.

#### 2.5.2. Cluster Labelling

Once the whole image has been coded, the next step is the labelling of the existing classes. If *M* thresholds haven been obtained, there are *n* = *M* + 1 histogram partitions per channel, and therefore the number of possible combinations is *n^d^*, where *d* is the number of spectral components, *i.e.*, *d* = 3 in the CIE L*a*b* colour space. This number of combinations represents the number of classes. Each cluster is identified by its label. Every pixel is assigned its corresponding label according to Equation (13). So, given the pixel *i* ≡ (*x*, *y*) with codes $\tilde{\iota_{L}}$, $\tilde{\iota_{a}}$, and $\tilde{\iota_{b}}$, its label will be given by *P̃_ι_* as follows:

(13) $${\tilde{p}}_{i}=n^{2}{\tilde{i}}_{L}+n{\tilde{i}}_{a}+{\tilde{i}}_{b}$$

#### 2.5.3. Merging Process

Let *C_k_* be the number of clusters obtained by the classification procedure, where *k* identifies a class between 1 and *n^d^*, each class containing *N_k_* pixels of the original image. It could be said that each class is defined by a tri-dimensional vector (*d* = 3). The elements of that vector are the spectral components of the pixels according to the CIELab colour model, *i.e.*, ${\text{i}}_{k}\equiv (i_{L}^{k},i_{a}^{k},i_{b}^{k})$ for the pixel *i* ≡ (*x*, *y*), if the pixel and its spectral components belong to class *C_k_*.

For each class, the average value of the membership degrees to that class is calculated by Equation (14):

(14) $$\mu_{k}\equiv \left(\mu_{L}^{k},\;\mu_{a}^{k},\;\mu_{b}^{k}\right)=\frac{1}{N_{k}}\sum_{i_{k}\in C_{k}}{\text{i}}_{k}$$

Based on the potential of Otsu’s method, it is possible to estimate the *within-class* and the *between-classes* spectral variances, denoted by *σ_k_* and *σ_kh_* respectively, according to Equations (15) and (16). Obviously, *σ_k_* is only related to class *C_k_* and, as expected, *σ_kh_* involves the two classes *C_k_* and *C_h_*, *k* ≠ *h*:

(15) $$\sigma_{k}=\frac{1}{d\cdot N_{k}}\sum_{{\text{i}}_{k}\in C_{K}}{\left[{\left(i_{L}^{k}-\mu_{L}^{k}\right)}^{2}+{\left(i_{a}^{k}-\mu_{a}^{k}\right)}^{2}+{\left(i_{b}^{k}-\mu_{b}^{k}\right)}^{2}\right]}^{1/2}$$

(16) $$\sigma_{kh}=\frac{1}{d}\left[{\left(\mu_{L}^{k}-\mu_{L}^{h}\right)}^{2}+{\left(\mu_{a}^{k}-\mu_{a}^{h}\right)}^{2}+{\left(\mu_{b}^{k}-\mu_{b}^{h}\right)}^{2}\right]$$

Based on those variances, some classes can be fused due to their spectral similarities. The similarity is a concept defined as follows. Given the classes *C_k_* and *C_h_*, *k* ≠ *h*, both are merged into one class if *σ_k_* ≥ *σ_kh_* or *σ_h_* ≥ *σ_kh_*. This is based on the hypothesis that if a good partition is already achieved, the classes obtained are properly separated, without overlapping, and then no further fusion is required. On the contrary, if classes overlap, the between-class variance *σ_kh_* is greater than the individual within-class variances, *σ_k_* and *σ_h_*. This re-clustering process is repeated until all the between-class variances are greater than their corresponding within-class variances. Without lost of generality, if two classes are merged, the resulting fused class will be re-labelled with the name of the class with the smaller variance value. This does not affect the classification process because only labels are modified.

After the fusion process, it must be checked if more clusters are necessary. This is carried out on the basis that if after the combination process no class has been fused, it means that more clusters are needed. A new clustering process starts again with the number of thresholds increased by one. This is repeated until a fusion occurs.

## 3. Results and Discussion

In order to show the performance of the proposed automatic unsupervised strategy, the images under study are briefly described and the new classification strategy is explained.

### 3.1. Oat Crop Description

The selected oat crop surface is 20 ha. Two thousand samples of 1 m^2^ were imaged by the CCD sensor. The images were taken two consecutive days in order to prevent significant changes in the plants. The weather conditions of these two days were different. One was a consistently sunny day and the other was a cloudy day. Moreover, the samples were obtained at different times of the day (morning, midday and afternoon). This was intended to verify the robustness of the proposed method against illumination variability.

**Figure 2.**
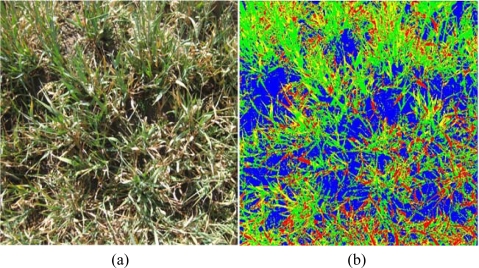
**(a)** Original digital image; **(b)** Classification results obtained by the unsupervised strategy (four re-clustering).

**Figure 3.**
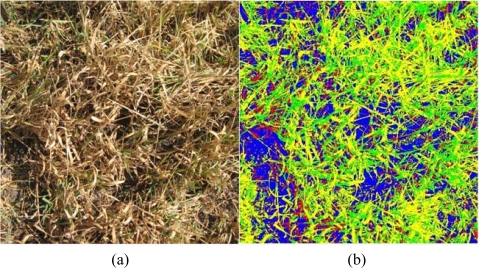
**(a)** Original image; **(b)** Classification results obtained by the unsupervised strategy (four re-clustering).

Two oat crop areas with quite different degrees of damage were selected; Figures 2(a) and 3(a) are representative samples of those areas. The degree of damage was assessed by an expert in agriculture and loss management.

1. **Description of the first area,**Figure 2(a): The green region density is 53.15% on average. The whole area is considered to show a low degree of frost damage. Some of the 1,000 samples of the healthy crop were used as reference data (10%) and the rest, *i.e.*, 900 images, were classified and compared to the test images.
2. **Description of the second area,** Figure 3(a): On average, the estimated green plant density is 26.6% and the rest are considered dried plants. Therefore this area is an example of high degree frost damage.

The aim of the classification process is to identify four classes depending on the degree of damage that due to very low temperatures. The first class is the healthy crop, which shows mostly green spectral components. The second cluster is the heavily damaged, dried crop, *i.e.*, mainly yellowish components. The third one represents an intermediate state, where the oat plants can be considered neither green nor yellow. The fourth class corresponds to shady ground. Plants belonging to the first group grow and develop regularly unless other stresses (e.g., frost or drought) affect them. Therefore, this group, once set in a certain number of kernels per square meter, is likely to fill them and produce certain yield levels. The second group does not contribute to the oat production. Experts estimate the usable harvested area of the third class at about 40–60%. Obviously, this quantification is calculated at the time of sensing, and therefore posterior damages are not considered.

Therefore, there are three different classes of oat plants textures that have to be recognized in order to identify the state of the crop; the fourth class is the shady ground. According to the procedure described in Section 2.5, one threshold is enough. As there are three components in the colour space, this will result in eight classes. The final solution will require a fusion of the eight classes into the four categories that correspond to the reality. This can be achieved by relaxing the merging criterion in order to force the fusion of the classes with the highest degrees of overlapping, which is determined by measuring the biggest difference between *σ_k_* or *σ_h_* and *σ_kh_*, Equations (15) and (16).

Figures 2(b) and 3(b) show the classification results of the original images presented in Figures 2(a) and 3(a), respectively. The labels of the four classes are represented by colours: (1) green for the first class; healthy oat (its real colour); (2) yellow for the dried plants; (3) red for the oat plants at an intermediate state between green and dried; (4) blue corresponds to shady ground category. The name of the classes are GO (Green oat), DO (Dried oat), HD (Half dried oat) and SG (Shady ground), respectively.

### 3.2. Validation of the Classifier

The validation of a classification process refers to the degree of concordance between the classes assigned to each pixel of the classified image and a set of reference data given by an expert. That is, if the classification agrees with the labelling of the expert. To obtain a quantitative estimation of the classifier performance, a fuzzy error matrix has been built [44,45]. This method has been proved as a good way to assess the validity of an image classifier. An error matrix is a square array of numbers that express the number of sample units (*i.e*., pixels or groups of pixels) assigned to a particular category by a classification process (rows) *vs*. sample units assigned to that particular category by another classification procedure that are taken as reference data (columns). In this case, the second classification procedure is the one carried out by the expert. The comparison between them gives the number of bad or good classified samples. Given the wide acceptance of the error matrix, an approach is used that combines both the error matrix and some measure of fuzziness, called the fuzzy error matrix approach, introduced by [45]. Both the size and the shape of the sample units that are going to be used have to be described before defining the reference data.

**Figure 4.**
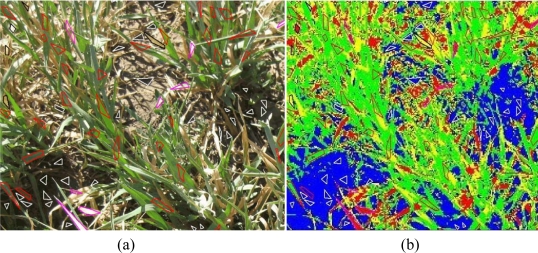
**(a)** Reference Data: sample units on the original image. **(b)** Sample units on the classified image.

- Selection of the Sample Units
  Historically, a single pixel has often been chosen as the sample unit. However, it is extremely difficult to know exactly where that pixel is located at the original image. Therefore, using a single pixel as sampling unit causes many of the errors represented in the fuzzy error matrix to be positional errors rather than thematic errors. Since the goal of the fuzzy error matrix is to measure thematic errors, it is better to take steps to avoid including positional errors. Based on the foregoing, polygons (groups of pixels, with different shape and size) have been chosen as sample units instead of single pixels. They are called polygons or sample units without distinction.
- Determination of the Number of Polygons or Sample Units
  There are several ways to estimate the minimum size of the polygons. It depends on the size of the image and the number of classes. In this case, a multinomial distribution has been applied [44]. The number of reference sample units is obtained by using the chi-square distribution, the desired confidence level, and the percentage of the image that is covered by each class, according to Equation (17):
  (17) $$n_{s}=B\Pi_{k}\;(1-\Pi_{k})/b_{k}^{2}$$
  where Π*_k_* is the fraction of the image surface of the image that belongs to class *k; B* is a coefficient obtained by the chi-square distribution with one degree of freedom and parameter *1* − *b*/*k*, *b* is the accuracy (in this case, *b* = 0.05 because the confidence degree has been set to 95%) and *k* is the number of classes or categories. For example, if there are four categories (*k* = 4), and the desired confidence level is 95% (b = 5%), it would be said that a particular class covers 16% of the image pixels (Π*_k_* = 16%). The value of *B* must be determined from a chi-square table with 1 degree of freedom and *1* − *b*/*k* = 1 − 0.05/4. In this case, the value of *B* is χ^2^(1, 0.9875) = 6.36640. Therefore, the number of sample units will be *n_s_* *=* 6.36640(0.16)(1 − 0.16)/0.05^2^ = 345. If the pixels of the image correspond to four classes, and each class covers 35%, 16%, 16% and 33% of the whole image, then the 345 polygons will be distributed into the four classes: 121, 55, 55 and 111, respectively.
- Sampling Scheme
  Once the number of polygons for each class has been determined according to Equation (17), the original image is analysed by an expert who assigns these polygons to their corresponding classes. This assignment is carried out based on the following criteria:
  - Each polygon should contain only pixels of one class; therefore the size and shape of each polygon must be adjusted to the objects of the image. For example, polygons related to classes involving oat leaves (majority in the analysed images) must have irregular shapes as shown in Figure 4.
  - The polygons depicted in the original image (Figure 4(a)) are known as reference data or ground truth data. An expert is asked to validate them.
  - The original images, with the polygons drawn, and the classified images are matched by using ArcView 3.2 GIS tool, considering that both images have identical UTM coordinates. So the corresponding polygons are drawn in the classified images and therefore the labels of the classes they belong to.

Figure 4 shows the distribution of these polygons for the shady ground category (white line), the green oat (red line), dried oat (black line) and intermediate state (pink line), on both images. The reference image has been labelled by the expert (Figure 4(a)), and Figure 4(b) is the image classified by the proposed strategy.

For any polygon in the classified image (Figure 4 (b)), whose location and label are both known, it is be possible to calculate the number of its pixels that have been correctly or incorrectly classified. This is the basis for computing the error matrices as described in the next section.

#### 3.2.1. Calculation of the Deterministic and Fuzzy Error Matrices

An error matrix is a very effective way to obtain and represent the classifier accuracy. The individual accuracies of each class are plainly described along with both the errors of inclusion (commission errors) and errors of exclusion (omission errors). A commission error is simply defined as including an area in a category when it does not belong to that class. An omission error is when a pixel is excluded from the category to which it belongs. We have compared 200 polygons out of the 2,000 samples. The results for each class are shown in Table 2, which represents simultaneously both the error matrix and the fuzzy error matrix.

The values of the main diagonal represent the number of correctly classified sample units. The off-diagonal cells contain a pair of values, separated by a semicolon. The first value represents those sample units that, although no absolutely correct, are considered as to be acceptably classified according to some fuzzy rules. The second value indicates those sample units that are unacceptably classified under the same fuzzy rules, *i.e*., they are considered as errors. For the deterministic matrix, those two values are added giving a unique value for incorrectly classified.

For example, the two values of the first row, third column are: 400 and 600. From the deterministic point of view, 1,000 sample units or polygons (400 + 600) have been wrongly classified into the GO category by our method, when they should have been classified as HD according to the reference data (columns). This means that 1,000 polygons are excluded from the correct HD category and included into the incorrect GO category. Under the fuzzy criterion, 400 polygons were considered acceptable classified and 600 sample units are unacceptable. The set of fuzzy rules to explain or capture the variation of this classification is the following:

- Absolutely correct: for a particular class, 100% of the surface of the reference polygons and the corresponding locations on the classified image overlap (major diagonal of the error matrix).
- Acceptable: at least 50% of overlap between the locations on the original image (reference) and that sample unit on the classified image for every class. For example, a polygon on the original image (reference) that belongs to class GO is projected into two classes on the classified image, GO and SG. But if at least 50% of that location matches up to the GO class, it is considered acceptable. This will add 1 to the counter at the left in the cell that corresponds to column GO, row SG.
- Error (unacceptable): more than 50% of a location that belongs to class *k* overlaps a different category. For instance, a polygon that in the reference image belongs to DO class, overlaps class GO on the classified image. This will add 1 to the value at the right of the cell (row GO, column DO).

In addition to clearly showing errors of omission and commission, the error and fuzzy error matrices can be used to compute other accuracy measures such as the overall accuracy, expert’s accuracy and classifier’s accuracy, which are of interest for agriculture inventories.

#### 3.2.2. Classifier’s Accuracy and Commission Errors

An overall accuracy level of 85% was adopted to represent the cut off between acceptable and unacceptable results. This standard was first described in 1976 by Anderson [46], and seems to be almost universally accepted [47]. The classifier accuracy represents the probability of a pixel to be correctly classified.

The deterministic (traditional) overall accuracy is simply the sum of the major diagonal (correctly classified sample units) divided by the total number of sample units in the entire error matrix, *i.e*., 63,400/68,800 = 92%.

The fuzzy assessment overall accuracy is estimated as the percentage of sites where the “good” and “acceptable” reference label(s) matched the classified image label; therefore the sum of the values along the major diagonal (absolutely correct sample units) and those deemed acceptable (first value in the off-diagonal) divided by the total number of sample units in the entire fuzzy error matrix, *i.e*., 66,200/68,800 = 96%.

The individual accuracy of each category is described together with the errors of inclusion (commission errors) in the classification. A commission error was defined as including a polygon of the classified image in a category when it does not belong to that class. The deterministic individual class accuracy is estimated by the major diagonal value (*i.e*., number of correctly classified sample units for this class) divided by the total number of classified locations; and the fuzzy assessment individual class accuracy is estimated by the sum of value the major diagonal (*i.e*., the correctly classified sample units for this class) and those deemed acceptable (*i.e*., the first value of each cell in the row) divided by the total polygons classified.

The values of this categories’ accuracy, for both the deterministic and fuzzy errors, are displayed in Table 3. The total, as it was said, is calculated by the values of the major diagonal (for the deterministic case) plus the non-diagonal first value of each cell (row), for the fuzzy assessment. Classifier accuracy is then obtained as Total/Total Classified. Commission error is computed as 100% minus Classifier’s accuracy for each class. The error values were taken from the results shown in Table 2.

For example, the classifier’s accuracy for the DO category is calculated by dividing the total number of correctly classified locations in that category (8,800 for the deterministic matrix and 9,400 for the fuzzy one) by the total number of polygons classified as dried oats (9,800, see Table 2). The value obtained is 90% and 96% for the deterministic and fuzzy cases respectively.

#### 3.2.3. Expert’s Accuracy and Omission Errors

The expert’s accuracy is calculated for every class, and it describes the ability to classify a particular category. This calculation is performed by dividing the total number of correct sample units in a particular category by the total number of sample units of that class as indicated by the reference data (*i.e*., 22,600/24,000 = 0.94 for GO). In this case, only the errors of exclusion (omission errors) are taken into account. An omission error means excluding an area from the category to which it belongs.

Table 4 shows the expert’s accuracy for the deterministic and fuzzy approaches when using the corresponding values of Table 2.

For example, the ability to classify DO can be obtained by dividing the total number of correctly classified sample units of this category (8,800 in the deterministic matrix, and 10,000 in the fuzzy one) by the total number of dried oat polygons as indicated by the reference data (10,800, column “total sample units”). This division results in an accuracy of 81% (deterministic) and 93% (fuzzy), which are quite good.

The Total columns of Table 4 represent the values of the major diagonal (for the deterministic approach) or these values plus the non-diagonal first value of each cell (column), for the fuzzy case. Then, Expert’s accuracy is obtained as Total/Total sample units. Omission error is the subtraction of the Expert’s accuracy from 100%.

#### 3.2.4. Accuracy and Errors for Simple and Combined Thresholding

Table 5 summarizes classifier and expert’s accuracy and commission and omission errors. Four thresholding approaches are compare: combined thresholding approach (CT), and the simple thresholding strategies Isodata (IS), Otsu’s (OT) and Fuzzy (FU), from both the deterministic and fuzzy points of view. The values presented in Table 5 are the average of the results for the four categories (GO, DO, HD, SG). As it can be seen in Table 5, the best performance is obtained by the proposed CT strategy in terms of both accuracy and error.

### 3.3. Summary and Discussion

To identify the level of damage in oat crops due to frost, an unsupervised classification strategy has been developed. It involves three main procedures: (a) an automatic thresholding; (b) fusion of thresholds and determination of frost damage using this merged value; (c) validation and accuracy estimation of the classifier by computing the error matrices.

The automatic thresholding is carried out by combining the three following thresholding strategies: Otsu’s, Isodata and Fuzzy. Combination of different classifiers has been proved to be useful to improve the classification results [43,48]. In this proposal, the average of these three thresholds for each spectral band of CIELab colour space is considered. For this agricultural application, the merging gives better results than the results gained when taking each of them separately.

The accuracy of a classification process refers to the degree of agreement between the classified image and the ground truth; to show quantitatively this accuracy, an error matrix has been calculated that allows us to identify some sources of confusion and not simply the “error”. The fuzzy error matrix was used to extend the results in order to consider uncertainty in the labelling. Each category can be analysed by studying the values of the rows. As shown in Table 2, the classes that have more classification errors are HD and DO. In this Table, the value of the third column (HD), last row (Total sample units), *i.e*., 11,200, stands for the number of sample units or polygons that were classified as HD by the expert. Our classifier assigned this category to 10,000 polygons (third row, last column). In the fuzzy error matrix it is possible to see that out of these 10,000, 9,200 were correctly classified (diagonal value). Furthermore, there are 200 sample units than were classified as HD when the reference data shows that they actually belong to the DO class. The other 600 sample units were classified as something between DO and HD, and therefore this classification was considered acceptable in a fuzzy way (see third row, second column, 600; 200). As the total number of classified sample units of this class is 10,000, the deterministic class accuracy is 92% (9,200/10,000), and 98% (9,800/10,000) for the fuzzy approach, Table 3.

The expert’s accuracy for the same category, HD, can be analysed taking into account the columns of Table 4. Out of 11,200 sample units classified as HD by the expert, 9,200 matched correctly, 600 were misclassified as GO, and 400 as DO. Regarding the fuzzy approach, 400 were located between GO and HD, and 600 between DO and HD, these classifications were considered acceptable. Therefore, the expert’s accuracy (Table 4), for the HD category is 82% (9,200/11,200), with an omission error of 18%, in the deterministic case. The fuzzy expert’s accuracy is 91% (10,200/11,200), with a fuzzy omission error of 9%. The same analysis can be done for every category.

These results allow the quantification of the oat crop damage due to very low temperatures. On 20 ha, an area of 7.6 ha has not been affected by frost. But low temperatures have partially damaged an area of 2.7 ha and caused a loss of 9.7 ha. Based on the protocol suggested by the SIAP [3] and using the historical data, it is possible to predict that 126.92 t ha^−1^ can still be produced from the healthy 7.6 ha.

## 4. Conclusions

A new unsupervised classifier has been designed that allows us to predict the quantification of the damage due to low temperatures on oats. The images are taken by a digital camera CCD sensor, *i.e*., with relative low cost. The CIELab colour model is used as it is closer to human perception and therefore more useful in agricultural applications.

The classification strategy is based on the fusion of three thresholding techniques. It generates as many classes as required by the application in an automatic way (called dynamic clustering, [49]). This flexibility on the number of classes is one of the main advantages of this method. The automatic classifier is able to correctly identify oats affected by very low temperatures (frost) from green oats and areas without vegetation (ground).

This methodology is useful for large-scale producers that can monitor their crops and thus to estimate the lost production. It is also useful for agricultural insurance purposes, as it facilitates the assessment of harvested areas. The same procedure could be applicable for quantifying the effects of pests, diseases, or droughts and therefore, extending the range of applicability.

Another agricultural application of such conventional sensors, that has not yet been developed, would be to assess the phenological state of short-cycle crops, such as oats, wheat and barley after being affected by weather.

An important contribution of this proposal, with respect to visual observations of experts, is the quantitative damage estimation. The decisions made by experts and growers, who are sometimes influenced by fatigue, are mainly based on qualitative aspects. This advantage justifies the use of the automatic vision system.

The implementation of this strategy to monitor crops could be quite useful. It will allow more accurate predictions on the production. It can also help to make a decision on the right treatment for the crop if necessary. Nevertheless, the application of this strategy to harvested areas should be done only after damage, as the changes in the oat crop are almost imperceptible day-to-day.

## Figures and Tables

**Table 2.**
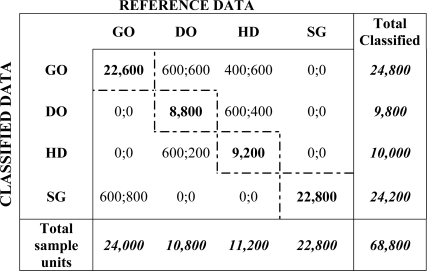
Deterministic and fuzzy error matrices.

**Table 3.** Classifier’s accuracy and categories’ accuracy (commission errors).

	
	**Deterministic**			**Fuzzy**		

**Classes**	**Total**	**Classifier’s accuracy**	**Commission errors**	**Total**	**Classifier’s accuracy**	**Commission errors**
**GO**	22,600	91%	9%	23,600	95%	5%
**DO**	8,800	90%	10%	9,400	96%	4%
**HD**	9,200	92%	8%	9,800	98%	2%
**SG**	22,800	94%	6%	23,400	97%	3%

**Table 4.** Expert’s accuracy and omission errors.

	
	**Deterministic**			**Fuzzy**		

**Classes**	**Total**	**Expert’s accuracy**	**Omission errors**	**Total**	**Expert’s accuracy**	**Omission errors**
**GO**	22,600	94%	6%	23,200	97%	3%
**DO**	8,800	81%	19%	10,000	93%	7%
**HD**	9,200	82%	18%	10,200	91%	9%
**SG**	22,800	100%	0%	22,800	100%	0%

**Table 5.** Classifier and expert’s accuracy and errors for the combined (CT) and simple (IS, OT, FU) thresholding approaches.

	
	**Deterministic**				**Fuzzy**			

	**Accuracy (%)**		**Errors (%)**		**Accuracy (%)**		**Errors (%)**	

	**Classifier**	**Expert**	**Commission**	**Omission**	**Classifier**	**Expert**	**Commission**	**Omission**
**CT**	91.78	89.45	8.22	10.55	96.44	95.08	3.56	4.92
IS	87.71	87.70	12.29	12.30	94.83	94.01	5.17	5.99
OT	85.67	83.10	14.33	16.90	90.99	88.20	9.01	11.80
FU	88.44	89.03	11.56	10.97	94.97	94.89	5.03	5.11
